# GC–MS Profiling of Naturally Extracted Essential Oils: Antimicrobial and Beverage Preservative Actions

**DOI:** 10.3390/life12101587

**Published:** 2022-10-12

**Authors:** Reham F. El-Kased, Dina M. El-Kersh

**Affiliations:** 1 Microbiology Department, Faculty of Pharmacy, The British University in Egypt, Cairo 11837, Egypt; 2 Pharmacognosy Department, Faculty of Pharmacy, The British University in Egypt, Cairo 11837, Egypt

**Keywords:** beverage preservative, natural essential oils, antimicrobial activity

## Abstract

The purpose of this study was to demonstrate the antimicrobial effects of natural essential oils (EO) and determine their preservative action. Eight natural essential oils were tested against *Staphylococcus aureus*, *Escherichia coli,* and *Candida albicans* representing gram positive, gram negative, and fungi, respectively. The plant materials were used in this study viz. *Thymus vulgaris*—thyme (TV), *Mentha virdis* (MV), *Mentha longifolia* (ML), *Rosmarinus officinalis*—rosemary (RO), *Lavandula dentata*—lavender (LD), *Origanum majorana*—oregano (OM), which belong to the Lamiaceae family. The other two plants were *Cymbopogon citratus*—lemon grass (family Poaceae) (CC), and *Eucalyptus globulus* (family Myrtaceae) (EG). Employing the disc diffusion susceptibility test, minimum inhibitory and minimum bactericidal concentrations were estimated for each oil, followed by the addition of oils to pasteurized apple juice after microbial induction. The results revealed that *thyme* oil showed the maximum zone of inhibition against all tested microbes enriched with monoterpenes class viz. eucalyptol (24.3%), thymol (17.4%), and γ-terpinene (15.2%). All other tested oils exhibited a concentration-dependent inhibition of growth and their MIC ranged from 0.1 to 100 µL/mL. The recorded minimum bactericidal concentration values were apparently double the minimum inhibitory concentration. The EO of *Mentha virdis* followed by *Mentha longifolia* showed maximum antimicrobial activity against the tested organisms in pasteurized apple juice. A gas chromatography–mass spectroscopy (GC–MS) analysis of lemon grass, thyme, and *Mentha virdis* essential oils showed their enrichment with monoterpenes class recording 97.10, 97.04, and 97.61%, respectively.

## 1. Introduction

Food poisoning is a widespread, life-threatening illness that is considered an important public health problem [1]. Its main cause is consuming food or beverages contaminated with viruses, bacteria, fungi, or their toxins. Foodborne disease incidence can be affected by climate factors such as temperature, humidity, and rainfall [2]. Eating raw vegetables, fruits, unpasteurized dairy, and raw meat or fish may increase the risk of contamination. On the other hand, some types of food might be contaminated by microbes through harvesting, improper storage, or even transport, which may lead to cross contamination upon improper cooking [3]. Mild cases of food poisoning may be improved without the need of drug treatments, others may be hospitalized. Pathogenicity of food poisoning illness may differ depending on the source of contamination and individual susceptibility. Population as immunocompromised, children, pregnant women, and the old aged, being more susceptible, show serious and life-threatening symptoms [4]. Food contaminating organisms may be bacteria, such as *Staphylococcus aureus* and *Escherichia coli*, or *Candida albicans* fungus [5]. Food contamination would be via infectious microorganisms and toxins at any stage of processing or production from the farm-to-table causing foodborne illnesses. Food poisoning symptoms appear hours or days after consuming contaminated food and frequently results in vomiting, nausea, watery or bloody diarrhea, and dehydration [6]. 

Gram-positive *Staphylococcus aureus (S. aureus)* bacterium normally exists as normal flora on the skin as well as mucosal membranes of healthy people. It is among the four most common bacteria causing foodborne diseases viz. *Salmonella*, *Clostridium perfringens,* and *Campylobacter*. *S. aureus* produces about 20 different types of enterotoxins [7]. *S. aureus* has the ability to resist some antibiotics such as methicillin resistance *S. aureus* (MRSA) [8]. *S. aureus* may lead to complicated gastrointestinal diseases with systemic progression [9]. 

Pathogenic *Escherichia coli* (*E. coli*) are gram-negative strains that are not affected by the acidity of stomach pH followed by the colonization in the intestine [10]. The environmental presence of *E. coli* in water and food, as natural reservoir, can widely lead to food contamination and therefore severe illnesses, including watery to bloody diarrhea with dehydration, gastro-intestinal upsets, and septicemia [11]. 

*Candida albicans* (*C. albicans*) is the most widespread fungus, causing systemic illnesses [12]. About 20 *Candida* species have been identified to cause serious infections in humans, especially in the immunocompromised [13]. *C. albicans* causes collateral damage to tissues thereby exacerbating the pathological effects of infections [14]. Consequently, the risk of devastating illnesses increases and can result in considerable mortality due to the persistence and worsening of some chronic inflammatory bowel diseases. 

Chemical preservatives were first used as food preservatives but with undesirable biological effects on humans while increasing microbial resistance [15].

The use of plant extracts and spices have been of great interest as a natural alternative to food preservatives. These natural substances have been used for seasoning, flavoring, and as food preservatives by preventing or inhibiting the growth of microorganisms [16,17]. In addition, they have been utilized for their antimicrobial, anti-inflammatory, and analgesic effects [18]. Many essential oils (EO) showed broad antimicrobial activity and could be used to prevent microbial contamination, guarantee safety, quality, and prolong shelf-life depending on their chemical composition which differ according to their plant origin [19]. Essential oils are categorized as safe (GRAS) by FDA and are more accepted by consumers than conventional chemical alternatives due to their natural origin [20]. 

Bacteria and fungi differ in their acquiring resistance to a specific plant extract. Effectively, natural plants have gained great interest in the food industry as important, natural additives to substitute chemical products [21]. These plants are traditionally used as natural plant remedies for various purposes, including treatment of infections, pain relief, decongestant, antifungal, antiviral, antioxidant, anti-inflammatory, and antiseptic [22].

The Lamiaceae family, (formerly Labiatae) has been attracting attention for the potent antimicrobial action of its plant species and therefore preservative effect. Being enriched with a high content of effective classes with antimicrobial action places the whole family as a powerful natural preservative agents. A recent review by Ramos de Silva, et al., 2021 discussed the importance of the Lamiaceae family as a potent antimicrobial, antioxidant, and other biological activities in context to their chemical profiling [23]. Algerian Thyme “*Thymus vulgaris*” was enriched with thymol ingredient, which varied as per its region in Algeria “59.5% and 67.7%”. *Origanum compactum* and *O. vulgare* were effective antimicrobial agents against *S. aureus* and *E. coli* gram-positive and negative bacteria, respectively. Both the Origanum species were chemically enriched with thymol, carvacrol, *p*-cymene, and *γ*-terpinene volatiles [23]. In a published study by Moumni et al., 2020, the authors worked on understanding the antimicrobial activity of some members of the Lamiaceae family cultivated in Tunisia with relation to their antimicrobial activity. The authors investigated the extracted EOs from *Rosmarinus officinalis*, *Thymus capitatus*, *Origanum majorana,* and *Salvia officinalis* by understanding their antimicrobial action on *Pseudomonas aeruginosa*, *Escherichia coli*, *Salmonella enterica*, *Bacillus subtilis,* and *Staphylococcus aureus*. The results proved the effectiveness of all tested EOs where the *T. capitatus* essential oil recorded the best results among other EOs. *T. capitatus* recorded MBC ranging from 0.73 to 2.94 mg/mL [24].

The aim of this study was to assess the antimicrobial as well as the preservative action of various naturally extracted essential oils by inhibiting the survival, growth, and multiplication of *S. aureus*, *E. coli,* and *C. albicans* present in fresh juice. The essential oils from different plant origins were extracted from members of the Lamiaceae family viz. Oregano, lavender, rosemary, lemon grass, *Mentha virdis*, *Mentha longifolia,* and thyme, in addition to Eucalyptus (Myrtaceae) and lemon grass (Poaceae) cultivated in Egypt. Further, in this study, the authors compared the activity of the extracted essential oils with limonene and eucalyptol selected as authentic volatiles being potent antimicrobial agents [25].

## 2. Materials and Methods

### 2.1. Plant Materials, Microorganisms, and a Prepared Juice Sample

#### 2.1.1. Plant and Standard Volatiles

Eight plants were used in this study, six belong to the Lamiaceae family viz. *Thymus vulgaris*—thyme (TV), *Mentha virdis* (MV), *Mentha longifolia* (ML), *Rosmarinus officinalis*—rosemary (RO), *Lavandula dentata*—lavender (LD), *Origanum majorana*—oregano (OM). The other two plants were *Cymbopogon citratus*—lemon grass (family Poaceae) (CC), and *Eucalyptus globulus* (family Myrtaceae) (EG). All previously mentioned plants have been purchased from the Experimental Station of Medicinal and Aromatic Plants, Pharmacognosy Department, Faculty of Pharmacy, Cairo University, Giza, Egypt, in July 2018. Voucher specimens have been deposited in the Faculty of Pharmacy Herbarium, The British University in Egypt. Voucher specimen codes are as follows: Thyme, TV-H01; *Mentha virdis*, MV-H02; *Mentha longifolia*, ML-H03; rosemary, RM-H04; lavender, LO-H05; oregano, OM-H06; lemon grass, CC-H07, and eucalyptus, EG-H08. Eng. Therese Labib, a consultant of plant taxonomy at the Egyptian Ministry of Agriculture, authenticated all the plants used in this current study. Limonene and eucalyptol standards were purchased from Sigma-Aldrich Chemie GmbH, Germany.

#### 2.1.2. Preparation of Volatile Oils

The dried grinded plants, each (500 g), were subjected to water distillation (750 mL) for 3–6 h using a Clevenger-type apparatus until plant exhaustion. For each plant, the extraction was carried out in triplets. The EOs yield and their chemical composition variation “in a quantitative not qualitative way” would vary, and so replicates of experimental procedures in EOs extraction and their GC–MS analysis would be encouraged to guarantee the reproducibility and data accuracy [26]. The yield of the volatile oils (expressed as volume mL/500 g weight) per each plant was 6.0 ± 0.10, 5.5 ± 0.21, 4.0 ± 0.12, 4.8 ± 0.11, 5.8 ± 0.14, 5.6 ± 0.30, 6.4 ± 1.20, and 5.0 ± 24 mL for the plant material (TV), (MV), (ML), (RO), (LD), (OM), (CC) and (EG), respectively. The obtained oils were placed in desiccator after collecting and kept at −4 °C in sealed vials the in dark for further analyses. The physical properties of EOs were assessed as per the Egyptian pharmacopoeia 1984 [27].

#### 2.1.3. Microorganisms

The microorganisms studied in this research represent frequent organisms involved in infections related to healthcare and food poisoning. Thus, clinically-pure strains of *Escherichia coli* representing gram-negative bacteria were isolated on Sorbitol MacConkey agar (Difco^TM^), *Staphylococcus aureus* representing gram-positive bacteria isolated on Mannitol salt agar (MSA) (Difco^TM^), and *Candida albicans* fungus isolated on Sabouraud Dextrose agar (Difco^TM^), all acquired from The British University in Egypt. 

#### 2.1.4. Juice Sample

Fresh apple juice (pH 6.5–7) was used to assess the preservative potential of the essential oils of the plants. Whole apple fruits were machine-squeezed and then underwent multiple filtration steps through filter paper until obtaining clear juice. Clear apple juice portions of 100 mL were placed in 250 mL bottles. This was followed by pasteurization of the juice by autoclaving at 70 °C for one minute followed by rapid cooling to 7 °C.

### 2.2. Methods of Analysis

#### 2.2.1. The Disk Diffusion Susceptibility Test

The disk diffusion susceptibility test was performed using Mueller-Hinton Agar (MHA) to assess the antibacterial and antifungal effects of the essential oils. Three concentrations of the essential oils were tested: 100% oil, 50% oil (diluted with ethanol with ratio 1:1), and 25% oil (diluted with ethanol with ratio 1:4). Four 6 mm sterile filter paper discs saturated with 20 μL of each of the dilutions were applied on plates on which each of the test organisms were streaked at a concentration equivalent to 0.5 MacFarland standard, and a disc impregnated with 20 μL solvent alone was used as a blank. A standard antibiotic disk was applied as a positive control. The antimicrobial activity was assessed by measuring the diameters of zones of inhibition after a period of incubation (18–24 h for bacteria and 48–72 h for *C. albicans*). Measuring the diameter of zones of inhibition in millimeters was performed using a Vernier caliper (together with the diameter of the disc). Three readings average were recorded. Zones of inhibition equivalent to or more than 7 mm reflected the antimicrobial activity of essential oils against the test organisms. An activity index (AI) of the tested essential oils was calculated, where the inhibition zone diameter of the tested essential oil was divided by that of the standard antimicrobial agent, and where an activity index greater than 0.5 was regarded as significant antimicrobial activity [28].

#### 2.2.2. Minimum Inhibitory Concentration (MIC) Determination

The MIC of essential oils was estimated by the broth dilution method for the selected test organisms which resulted in diameters of inhibition zones of more than 7 mm. Bacterial and fungal test strains dilutions were prepared using Mueller Hinton Broth (Difco^TM^ Detroit, MI, USA) and Sabouraud Dextrose Broth (Difco^TM^, Detroit, MI, USA), respectively. Five 2-fold serial dilutions of essential oils were prepared with the highest and lowest concentrations of 2000 μL·mL^−1^ and 125 μL·mL^−1^ for bacteria and fungi. The final volume of the prepared concentrations was adjusted to the number of the test organisms. Standard antibiotics (Clotrimazole against fungi and Ofloxacin against gram-positive and gram-negative bacteria) were prepared using the same procedure. To each of the dilution and control tubes containing broth only, a standard inoculum (1.5 × 10^8^ CFU·mL^−1^) was added to reach final highest and lowest concentrations of 1000 and 62.5 μg·mL^−1^ for bacteria and 10,000 and 625 μg·mL^−1^ for fungi. After the incubation time (18–24 h for bacteria, 5–10 days for fungi), the test tube with the least concentration of essential oils with no visible growth was regarded as the MIC against the test microbe. An average of three readings was recorded as MIC. In this study, a MIC of less than 100 μL·mL^−1^ was considered as good antimicrobial activity, MICs of 100–500 μL·mL^−1^ with moderate activity, MICs of 500–1000 μL·mL^−1^ with weak activity, and MICs greater than 1000 μL·mL^−1^ with no activity [28].

#### 2.2.3. Minimum Bactericidal Concentration (MBC) and Minimum Fungicidal Concentration (MFC) Determination 

MBC and MFC determination were performed by taking 0.1 mL from MIC tubes showing no observable growth was inoculated onto Mueller Hinton agar (Difco^TM^, Detroit, MI, USA) and Sabouraud Dextrose agar (Difco^TM^, Detroit, MI, USA) for fungi by the spread plate method. At the end of incubation time (18–24 h for bacteria and 5–10 days for fungi), the lowest concentration of essential oils with no observable growth on subculture was regarded as its MBC and MFC against the test microbe. The ratios of MFC:MIC or MBC:MIC were estimated to determine the antifungal or antibacterial activity of essential oils against the test microbes, respectively. The compound is bactericidal or fungicidal when the ratio is between 1:2 to 2:1, and it is bacteriostatic or fungistatic if the ratio is greater than 2:1 [28].

#### 2.2.4. Induction of Microorganisms in Juice

The concentrations of the 3 different microbial strains under investigation were adjusted to 1.5 × 10^8^ CFU.mL^−1^ (0.5 McFarland) and each were grown in its relevant media. One mL of each microbial suspension was added to 4 mL sterilized apple juice to form a total volume of 5 mL in the sterile falcon tube. This step was immediately followed by the addition of the essential oils under investigation at concentrations according to their minimum inhibitory concentration (MIC) obtained values and stored at room temperature (Supplementary Table S1). Two control tubes were prepared, one containing juice and microbial strain only and the other contained juice only.

Rigorous antiseptic measures were applied throughout the sample preparation and inoculation to prevent any possible microbial contamination. 

#### 2.2.5. Bacterial Count/Viable Count

The original sample was diluted so that a range of 30 to 300 colonies of the test bacterium are grown. A number of dilutions were cultured to be certain that a suitable number of colonies will be grown (500 μL, 200 μL, and 100 μL). Serial dilutions of the sample in sterile water were performed (1:10, 1:100, 1:1000 etc.). This was followed by cultivation on a nutrient agar dish then sealed and incubated. The media used include nutrient agar for the *S. aureus* count or MacConkey agar to count *E. coli* gram-negative bacteria or Sabouraud Dextrose agar for *C. albicans*. One set of dishes were incubated at 22 °C for 24 h and a second set at 37 °C for 24 h. Colonies are counted by eye at the end of the incubation time. 

#### 2.2.6. GC–MS analysis of Essential Oils

An analysis of each essential oil was carried out separately via gas chromatography-mass spectrometry (GC-MS) where the procedure was adopted from a previous work [29]. A system operating Shimadzu GCMS-QP2010 (Tokyo, Japan) was used with the following conditions: The column (RTX-5 MS) was used with specifications that were (30 m × 0.25 mm i.d. × 0.25 µm film thickness) (Restek Corporation, Bellefonte, Pennsylvania, USA). The starting temperature of the column was 45 °C for 2 min and then increased to 300 °C at a rate of 5 °C/min and kept steady for 5 min. The temperature of the injector was 250 °C. The flow rate of helium (carrier gas) was (1.41 mL/min). The following conditions were applied when recording the mass spectra: (equipment current) filament emission current, 60 mA; ionization voltage, 70 eV; ion source, 200 °C. Automatic injection of the essential oil was at (1 µL, 1% *v*/*v*) with a splitting ratio (1:15). The identification of volatile metabolites was performed upon comparing the mass spectra as well as the retention index with those of the National Institute of Standards and Technology’s (NIST) chemistry webbook library. In addition, literature data was used to identify *n*-alkanes series by comparing their mass spectra and retention indices.

## 3. Results

### 3.1. The Disk Diffusion Susceptibility Test

**Figure 1:**
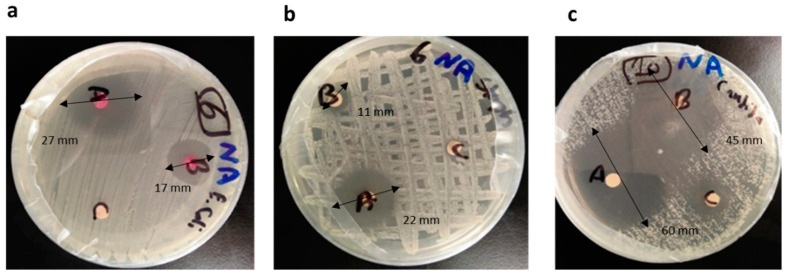
The disc diffusion susceptibility test of thyme against (**a**) *E coli.* (**b**) *S. aureus* and (**c**) *C. albicans.* A: 100% oil concentration, B: 50% oil concentration, and C: ethanol as negative control.

The disk diffusion method was used to test the antibacterial and antifungal activities of the essential oils against the selected microorganisms: *E. coli* as gram-negative bacteria, *S. aureus* as gram-positive bacteria, and *C. albicans* representing fungi. The oils were tested in 3 concentrations: 100%; 50%, and 25%. The antibacterial and antifungal activities were estimated according to the American Society for Microbiology, where zones of diameter < 12.00 mm were considered resistant, zones of diameter ranging 13.00–14.00 mm were considered with intermediate activity, and zones of diameter more than 15.00 mm were considered susceptible. The tested oil concentrations showed variable activities according to the results shown in Table 1 and Figure 1. The results showed that thyme (100% and 50% concentrations) and limonene (all concentrations) showed the maximum activity against *E. coli*, whereas thyme (100% concentration) and lemon grass (all concentrations) showed maximum activity against *S. aureus*. Against *C. albicans*, the oils showed decreasing activity according to the following order: thyme (100% and 50% concentration), limonene (100% and 50% concentration), *Mentha virdis* (100% concentration) and lemon grass (100% concentration), while 25% limonene and 50% lemon grass were the same in showing the least activity.

### 3.2. Minimum Inhibitory Concentration (MIC) Determination

The MIC of oils was determined against the selected strains (*E. coli, S. aureus,* and *C. albicans*) where the oils exhibited concentration-dependent inhibition of growth and the MIC of oils ranged from 0.1 to 100 µL/mL as shown in Table 2. The table shows that the least MIC (0.1 µL/mL) against *E. coli* was shown by *lemon grass* whereas the highest MIC (25 µL/mL) was shown by *oregano* and *lavender*, whereas against *S. aureus,* the least MIC (0.2 µL/mL) and the highest MIC (100 µL/mL) were shown by *lemon grass* and *limonene,* respectively. *Lemon grass* and *thyme* showed the least MIC against *C. albicans* (0.8 µL/mL) and the highest MIC (12.5 µL/mL) was shown with *Mentha longifolia*. 

Minimum bactericidal concentration (MBC) and minimum fungicidal concentration (MFC):

The results in Table 2 shows that the least MBC against *E. coli* was with *lemon grass*, *Mentha longifolia* and *rosemary* at concentrations 0.2 μL/mL, 0.4 μL/mL, and 0.8 μL/mL, respectively, whereas *Mentha virdis*, *thyme* and *limonene* showed the highest MBC (3.2 μL/mL). Whereas against *S. aureus,* the least MBC was 0.4 μL/mL and 3.2 μL/mL with thyme and lemon grass, respectively, whereas MBC was highest with Eucalyptus (50 μL/mL). Regarding *C. albicans,* the least MBC (1.6 μL/mL) was determined with *thyme* and *lemon grass*, whereas *oregano*, *rosemary* and *lavender* appeared to have no fungicidal activity at all. Table 2 also shows that the recorded MBC values were apparently double the MICs.

### 3.3. Bacterial Count/Viable Count

Oils were added to apple juice samples according to their calculated MIC. Oils added to apple juice samples containing *E. coli* showed a decline in the number of bacteria in decreasing order: *Mentha virdis*, *Mentha longifolia* and *limonene*. Regarding the apple juices containing *S. aureus* and *C. albicans*, *Mentha virdis*, *Mentha longifolia,* and *Limonene* showed an increased number of bacteria at day 1, which declined by days 5 and 7 to less than 30 CFU (Supplementary Table S2). Other oils did not show any remarkable activity where the bacterial load increased to more than 300 CFU by time. In this study, pasteurized apple juice was expected to have a good initial microbiological quality. All essential oils concentrations added to the juice were selected according to the calculated MIC for each oil. All experiments were assessed at room temperature where the sensitivities of *E. coli*, *S. aureus,* and *C. albicans* to essential oils were in the following decreasing order: *Mentha virdis*, *M. longifolia.* and limonene. 

### 3.4. Physical and Chemical (GC-MS) Analyses of Essential Oils

The physical properties “specific gravity, relative density, refractive index” in addition to oil appearance, color, and odor of EOs of *Cymbopogon citratus* “lemon grass”, *Thymus vulgaris* “thyme” and *Mentha virdis* “mentha” volatiles have been investigated and summarized in Table 3.

**Figure 2:**
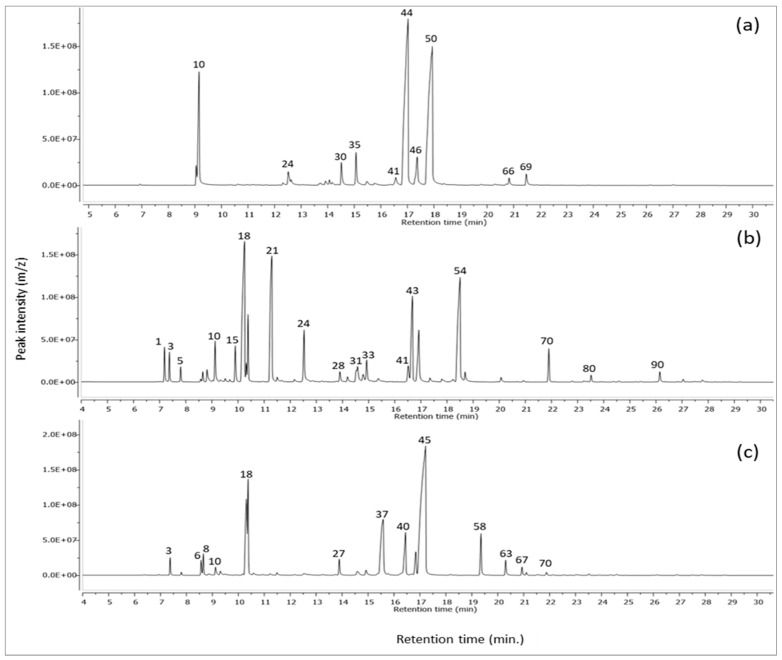
GC–MS chromatogram of volatile oil of (**a**): *Cymbopogon citratus* “lemon grass”, (**b**): *Thymus vulgaris* “thyme” and (**c**): *Mentha virdis* “mentha” herbs.

Upon an GC–MS chemical analysis of volatile oils prepared from *Cymbopogon citratus* (Poaceae), *Thymus vulgaris* (Lamiaceae), and *Mentha virdis* (Lamiaceae), a total of 24, 51, and 43 compounds have been identified in *Cymbopogon citratus*, *Thymus vulgaris,* and *Mentha virdis* volatile oils, respectively. It was observed that in all prepared essential oils, the major class was monoterpenes followed by sesquiterpenes. In both *Cymbopogon citratus* and *Mentha virdis* oils, the monoterpenes percentiles were 97.41% and 97.61%, respectively, followed by *Thymus vulgaris* 93.04%. The total sesquiterpenes in the three analyzed oils were 4.93, 2.4, and 0.89% for *Thymus vulgaris, Mentha virdis* and *Cymbopogon citratus* herbs, respectively. In *Cymbopogon citratus* oil, the monoterpenes class exemplified by dominance of geranial 36.35%, followed by neral 35.00%, representing monoterpene aldehydes, then *β*-myrcene monoterpene hydrocarbon 11.7% as presented in Figure 2a and Table 4. These results complied with previous studies representing almost the same percentiles where the identification of those monoterpenes as majors in the volatile oil of *Cymbopogon citratus* herb cultivated in Cameroon [30]. In a previous article, it was proved the potential efficacy of *Cymbopogon citratus* oil and its main citral “Geranial” being a potent antimicrobial agent against polymicrobial biofilm forming bacteria *viz.* Staphylococcus aureus and Candida species. The underlying mechanism of action was refereed to its citral volatile ingredient through reducing the biofilm mass and cell viability by interfering of nucleic acids, proteins, and carbohydrates of the biomass that lead to deformity of the biomass matrix in addition to disruptions to the biomass adhesive characters. It worth mentioning that the enrichment of the Egyptian *Cymbopogon citratus* oil with the citral volatile ingredient of this current study (36.35%) than that mentioned in Gao, et al., 2020 (29.364%) [31]. In *Thymus vulgaris* volatile oil, the identified major monoterpenes were eucalyptol as monoterpenoid oxide 24.30%, thymol monoterpenoid phenol 17.40%, and γ-terpinene monoterpene hydrocarbon 15.20% shown in Figure 2b and Table 4 where similar percentiles of the major monoterpene class except eucalyptol have been recorded in the previous literature [32]. The enrichment of *Thymus vulgaris* volatile oil with eucalyptol 24.30% counted for its potent antimicrobial activity against a myriad of pathogens as previously mentioned [33]. In a published article by Sienkiewicz et al., 2011, the authors tested the antimicrobial activity of Thymus vulgaris oil against a myriad of clinically multidrug resistant strains of *Staphylococcus, Enterococcus, Escherichia,* and *Pseudomonas* genus. The results revealed the strong antimicrobial activity of the tested thyme oil against multidrug resistant microbes previously mentioned. The authors would relate the bioactivity of thyme oil to its main volatile ingredients *p*-cymene and thymol recording 29.10 and 38.10%, respectively [34]. The GC–MS analysis of the Egyptian Thymus vulgaris oil in this current study revealed its enrichment with eucalyptol (1,8 cineole) by (24.3%), in addition to thymol (17.4%). Eucalyptol itself possessed potent antimicrobial activity as reported in previous works [34,35]. The volatile oil of *Mentha virdis* enriched with mainly carvone “monoterpene ketone” 42.50% followed by eucalyptol “monoterpene oxide” 17.40% then finally dihydrocarveol “monoterpene alcohol” 13.00% as in Figure 2c and Table 4 where these data matched with the previous literature by Mkaddem et al., 2022 [36] upon which an analysis of the oil obtained from *Mentha virdis* collected from Tunisia. In a previous article by Mkaddem et al., 2022, *Mentha virdis* oil was rich with carvone (50.47%), eucalyptol (9.14%), and limonene (4.87%) which encountered the potent antimicrobial activity of the oil against *Listeria monocytogenes* and *Klebsiella pneumoniae* bacteria [36]. It is worth mentioning in this previous study that despite the enrichment of *Mentha virdis* oil by carvone up to 50.47%, still the oil obtained from the Egyptian *Mentha virdis* was richer with other oxygenated monoterpenes “eucalyptol 17.40% and dihydrocarveol 13.00%” than the Tunisian one. The major volatile structures of each oil are illustrated in Supplementary Figure S1.

## 4. Discussion

*E. coli*, *S. aureus,* and *C. albicans* are common pathogens causing serious systemic infections in humans. Since the continuous development of antimicrobial resistance, natural products and essential oils have been studied as alternatives for the treatment of infections acquired in healthcare [16,37,38]. 

Essential oils are important sources of new antimicrobial agents particularly against bacterial pathogens. In vitro studies in this research demonstrated that the tested essential oils inhibited microbial growth with variable effectiveness [31]. 

The tested oils exhibited concentration dependent inhibition of growth and the MIC of oils ranged from 0.1 to 100 µL/mL. MBC/MFC was assessed to demonstrate the least concentration of oils resulting in microbial viability reduction of 99.90% of the initial count. The MBC readings were double the MICs, which indicates that the bactericidal activities of the oils occur at concentrations higher than its growth inhibitory concentrations.

It has been observed the enrichment of the analyzed volatiles of lemon grass, thyme and mentha with oxygenated monoterpenes mainly geranial and neral with a total percent of 74.34%, eucalyptol and thymol with total amount of 41.70% whereas carvone, eucalyptol, and dihydrocarveol with a total content of 72.80%, respectively. Volatile oils enriched with oxygenated monoterpenes encountered the oil as being more potent as antimicrobial rather than monoterpene hydrocarbons [39]. Allenspach et al., 2020 in a recent article for absolute quantification of terpenes in conifer species, the authors implemented a validated simple method for quantification of different terpenes viz. α and β-pinenes, camphene, 3-carene, limonene, bornyl acetate, β-caryophyllene, and borneol. Antibacterial activity of conifer essential oil proved its efficacy on both *E-coli* and *S. aureus* as gram-negative and positive bacteria, respectively [40]. In this current study, the GC–MS analysis proved the presence of such terpenes although in low percentile as listed in Table 3, still may be related to the antibacterial activity of the tested oils in this study. It is worth mentioning that previous studies worked on the mechanism of actions being antibacterial for each component in the volatile oil. In a previous work by Oz, et al., 2015, it was mentioned that the presence of an aromatic ring and a polar functional group of a volatile constituent “thymol” could lead to rupture of the bacterial cell membrane leading to release of the vital cell constituents [41]. A previous published review by Wińska et al., 2019 mentioned the effectiveness of essential oils as antimicrobial agents, specifically thyme and mentha in context to their volatile chemical profiling and major phytochemical ingredients. The antimicrobial activity of thyme would be referred to its enrichment with thymol (36–55%) and *p*-cymene (15–28%) whereas mentha antimicrobial activity referred to its higher percentile of menthol (30–55%) and menthone (14–32%) [42]. Citral “neral and geranial isomers”, have been approved by the U.S Food and Drug administration as being safe, so its use as natural preservative and flavoring agent due to its antibacterial activity against gram-negative and positive bacteria as *Escherichia coli* and *Staphylococcus aureus*, respectively [43,44]. Eucalyptol “1,8 cineole” antibacterial mechanism was summarized in a previous review where its effect mainly was due to changes in both size and shape of gram- positive and gram negative bacterial cell which ends with apoptosis [45]. α-pinene was detected in both thyme and mentha oils ca. 1.68 and 1.2%, respectively. In a recent published review by Allenspach and Steuer, 2021, the authors summarized the different biological activities of α pinene, of which they mentioned its positive antimicrobial activity on both gram-positive and negative bacteria *viz.* methicillin-resistant Staphylococcus aureus (MRSA), *Escherichia coli* (*E. coli*), and antifungal activity against Candida species [46]. Concerning carvone antibacterial mechanism of action, in a previous study, carvone enriched in the oil of *Mentha spicata* causes instability of phospholipid bilayer structure as well as interacts between the bacterial membrane enzymes and proteins [47]. 

Finally, the volumes of oils added ranged from 0.8 μL to 400 μL in 5 mL juice (with a range from 0.016% to 8%) (Supplementary Table S1). Concerning the taste of drinks after the addition of the EOs; EOs improve the flavor, odor, and color when added to foods. Many individual EOs are approved food flavorings and impart a certain flavor to foods, as well as delaying food spoilage without changing the organoleptic properties of the food. However, certain strategies could be implemented to decrease the organoleptic effects, if found, by optimizing the food/beverage formulation or by combining the essential oils and/or their active constituents with other means of sterilization such as pH or heat treatment (when applicable) [48]. 

## 5. Conclusions

Chemical preservatives have been utilized as food preservatives, but they turned to have undesirable biological effects on humans and increase in microbial resistance. This study demonstrates that natural volatile oils extracted from plants exhibited a concentration-dependent inhibition of microbial growth and offer potential antimicrobial activity against common food spoilage bacteria and fungi. For future research, the authors would encourage a larger scale comparative study of natural essential oils to chemical ones commonly used in the market as well as preparing a commercial naturally preserved effective product to replace chemical preservatives.

## Figures and Tables

**Table 1:** Measurements of zones of inhibition showing the activity of different oils concentrations against the tested microorganisms.

Oil/ Microorganism		*E. coli*	*S. aureus*	*C. albicans*
***Mentha virdis***	**Oil Concentration (%)**	**Zones of Inhibition (mm)**		
	100	7 ± 0.42	10 ± 0.60	29.5 ± 1.50
	50	6 ± 0.21	12 ± 0.80	7 ± 0.58
	25	6 ± 0.23	8 ± 0.70	11.5 ± 0.95
**Lavender**	100	5 ± 0.12	-	-
	50	6.5 ± 0.22	-	-
	25	5.5 ± 0.13	-	-
**Rosemary**	100	6 ± 0.22	13 ± 1.20	-
	50	6 ± 0.40	8 ± 0.90	-
	25	2 ± 0.0	9 ± 0.80	-
**Eucalyptus**	100	6 ± 0.20	12 ± 0.99	12.5 ± 1.00
	50	10 ± 0.3	-	9 ± 0.95
	25	13 ± 0.35	-	8 ± 0.55
**Thyme**	100	27 ± 1.5	22 ± 1.20	60 ± 1.50
	50	17 ± 1.0	11 ± 0.99	45 ± 1.00
	25	3 ± 0.003	-	-
**Lemon grass**	100	12 ± 0.90	40 ± 1.20	25 ± 0.80
	50	13 ± 0.90	20 ± 1.20	16 ± 0.95
	25	9 ± 0.60	18 ± 1.10	10 ± 0.55
***M. longifolia***	100	6 ± 0.30	4 ± 0.00	9 ± 0.00
	50	6.5 ± 0.29	-	8 ± 0.85
	25	7.5 ± 0.30	10 ± 0.70	8 ± 0.95
**Oregano**	100	7 ± 0.30	-	-
	50	6 ± 0.28	8 ± 0.31	-
	25	8 ± 0.31	-	-
**Limonene**	100	36 ± 2.82	-	36 ± 1.20
	50	34 ± 1.22	10 ± 0.68	34 ± 1.30
	25	16 ± 1.10	-	16 ± 0.95

**Table 2:** Minimum inhibitory concentration (MIC), minimum bactericidal concentration (MBC), minimum fungicidal concentration (MFC), and MIC:MBC ratio of oils against *E. coli, S. aureus,* and *C. albicans*. ND: not defined; NC: not calculated.

Oils	*E. coli*			*S. aureus*			*C. albicans*		
	MIC (µL/mL)	MBC (µL/mL)	MBC: MIC	MIC (µL/mL)	MBC (µL/mL)	MBC: MIC	MIC (µL/mL)	MBC (µL/mL)	MBC: MIC
*Mentha virdis*	1.6 ± 0.02	ND	NC	12.5 ± 0.85	ND	NC	3.2 ± 0.85	ND	NC
Lavender	25 ± 1.20	ND	NC	NC	NC	NC	NC	NC	NC
Rosemary	4.0 ± 0.03	ND	NC	NC	NC	NC	NC	NC	NC
Oregano	25 ± 0.95	ND	NC	6.3 ± 0.55	ND	NC	NC	NC	NC
Eucalyptus	6.3 ± 0.80	ND	NC	25 ± 1.10	ND	NC	6.3 ± 0.95	ND	NC
Thyme	1.6 ± 0.40	ND	NC	1.6 ± 0.25	3.2	2:1	0.8 ± 0.05	1.6	2:1
Lemon grass	0.1 ± 0.00	0.2	2:1	0.2 ± 0.00	0.4	2:1	0.8 ± 0.06	1.6	2:1
*M. longifolia*	0.2 ± 0.00	0.4	2:1	50 ± 1.95	ND	NC	12.5 ± 1.25	ND	NC
Limonene	1.6 ± 0.45	ND	NC	100 ± 2.55	NC	NC	1.6 ± 0.85	ND	NC

**Table 3:** The physical properties of essential oils (average of 3 independent experiments ± SD) of *Cymbopogon citratus* “lemon grass”, *Thymus vulgaris* “thyme” and *Mentha virdis* “mentha” volatiles.

Physical Property	EOs Yield (mL/500 g Plant)	Specific Gravity (25 °C)	Relative Density (g/cm^3^)	Refractive Index (20 °C)	Appearance	Color	Odor
**Lemon grass oil**	6.4 ± 1.2	0.9012 ± 0.03	0.893 ± 0.01	1.4862 ± 0.16	Clear oil	Pale yellow	citrus
**Thyme oil**	6.0 ± 0.10	0.9180 ± 0.04	0.865 ± 0.06	1.4823 ± 0.15	Clear oil	Pale yellow	thyme
**Mentha oil**	5.5 ± 0.21	0.9160 ± 0.01	0.910 ± 0.08	1.4638 ± 0.11	Clear oil	Pale yellow	mentha

**Table 4:** GC–MS analysis of essential oils (average of 3 independent runs ± SD) of *Cymbopogon citratus* “lemon grass”, *Thymus vulgaris* “thyme” and *Mentha virdis* “mentha” volatiles. Volatiles are listed regarding their elution on GC column (RTX-5). **RT**: retention time (min), **KI obs**.: Kovat’s index observed practically (RTX-5 GC column) related to C_8_-C_28_ n-alkanes series. KI. Lit.: Kovat’s index reported from the previous literature. MS: The volatile components identification was based on mass spectral data. KI: The volatile components identification was based on comparing KI published in mass spectral library of National Institute of Standards and Technology (NIST). Bold values are the major constituents in the volatile oil.

Peak No.	Rt. (Min.)	Name of Compound	KI (Obs.)	KI (Lit.)	Area (%)			Chemical Class	Identification
					Lemon Grass Oil	Thyme Oil	Mentha Oil		
1	7.17	β-Thujene	910	902	_	2.09 ± 0.720	_	Monoterpene	MS, KI
2	7.19	α-thujene	910	905	_	_	0.04 ± 0.001	Monoterpenes	MS, KI
3	7.37	α-pinene	917	917	_	1.68 ± 0.0320	1.2 ± 0.030	Monoterpenes	MS, KI
4	7.72	2,4(10)-Thujadiene	930	946	_	0.04 ± 0.0050	_	Monoterpene	MS, KI
5	7.81	Camphene	933	929	_	0.86 ± 0.020	0.21 ± 0.004	Monoterpenoids	MS, KI
6	8.57	Sabinene	961	964	_	_	1.07 ± 0.560	Monoterpenes	MS, KI
7	8.58	4(10)-Thujene	961	969	_	0.19 ± 0.001	_	Monoterpene	MS, KI
8	8.65	β-pinene	964	965	_	0.62 ± 0.050	1.54 ± 0.580	Monoterpenes	MS, KI
9	8.82	Vinyl amyl carbinol	970	969	_	1.23 ± 0.063	_	Alkenyl alcohol	MS, KI
10	9.16	β-Myrcene	982	985	**11.69 ± 1.300**	3.36 ± 0.730	0.94 ± 0.020	Monoterpenes	MS, KI
11	9.33	3-octanol	988	988	_	0.15 ± 0.003	_	Aliphatic alcohol	MS, KI
12	9.49	pseudolimonene	994	996	_	_	0.3 ± 0.002	Monoterpenes	MS, KI
13	9.51	a-Phellandrene	995	994	_	0.22 ± 0.002	_	Monoterpene	MS, KI
14	9.69	3-Carene	1001	1004	_	0.14 ± 0.003	_	Monoterpene	MS, KI
15	9.9	4-Carene	1008	1011	_	2.53 ± 0.430	_	Monoterpene	MS, KI
16	9.9	2-carene	1008	1010	_	_	0.07 ± 0.001	Monoterpenes	MS, KI
17	10.17	O-cymene	1017	1021	_	_	0.12 ± 0.001	Monoterpenes	MS, KI
18	10.39	Eucalyptol	1024	1020	0.05 ± 0.010	**24.30 ± 3.530**	**17.40 ± 3.220**	Monoterpene oxide	MS, KI
19	10.59	trans-β-ocimene	1030	1042	_	_	0.19 ± 0.030	Monoterpenes	MS, KI
20	10.93	β-ocimene	1041	1046	0.26 ± 0.020	0.04	0.07 ± 0.005	Monoterpenes	MS, KI
21	11.23	γ-terpinene	1051	1055	_	**15.2 ± 1.720**	0.17 ± 0.020	Monoterpenes	MS, KI
22	11.49	trans-sabinene hydrate	1059	1051	_	1.2 ± 0.040	0.33 ± 0.060	Monoterpenes	MS, KI
23	11.67	Linalool oxide	1065	1071	_	0.28 ± 0.002	_	Monoterpene	MS, KI
24	12.52	β-linalool	1092	1098	1.58 ± 0.340	4.79 ± 0.050	0.64 ± 0.030	Monoterpenes	MS, KI
25	12.63	Cis-verbenol	1096	1095	0.90 ± 0.030	_	_	Monoterpenes	MS, KI
26	13.74	Pinen-3-ol	1131	1131	_	_	0.12 ± 0.006	Monoterpene ketone	MS, KI
27	13.89	Camphor isomer	1136	1141	_	_	1.50 ± 0.400	Monoterpenoid ketone	MS, KI
28	13.9	Camphor	1137	1131	0.40 ± 0.010	0.97 ± 0.050	_	Monoterpene ketone	MS, KI
29	14.19	Isomenthone	1146	1148	_	0.60 ± 0.030	_	Monoterpenoid	MS, KI
30	14.52	t-verbenol	1156	1148	2.27 ± 0.010	_	_	Monoterpenes	MS, KI
31	14.59	Isoborneol	1056	1159	_	2.5 ± 0.060	0.86 ± 0.300	Monoterpene alcohol	MS, KI
32	14.79	Menthol	1165	1164	__	0.88 ± 0.040	_	Monoterpenoid	MS, KI
33	14.92	Terpinen-4-ol	1170	1177	_	2.19 ± 0.060	0.77 ± 0.030	Monoterpene alcohol	MS, KI
34	15.1	Levomenthol	1175	1172	_	0.03 ± 0.005	_	Monoterpenoid	MS, KI
35	15.13	Isoneral	1174	1174	2.91 ± 0.02	_	_		MS, KI
36	15.39	α-Terpineol	1185	1189	_	0.56 ± 0.005	_	Monoterpene alcohol	MS, KI
37	15.58	Dihydrocarveol	1191	1202	_	_	**12.9 ± 2.600**	Monoterpene alcohol	MS, KI
38	15.61	Trans-Dihydrocarvone	1192	1195	_	0.14 ± 0.003	0.35 ± 0.030	Monoterpenoid	MS, KI
39	15.764	Decanal	1197	1207	0.42 ± 0.020	_	_	Monoterpenes	MS, KI
40	16.45	trans-carveol	1220	1219	_	_	5.99 ± 1.600	Monoterpene alcohol	MS, KI
41	16.518	Citronellol	1223	1228	1.23 ± 0.040	1.62 ± 0.040	_	Monoterpenoid	MS, KI
42	16.84	carveol	1234	1246	_	_	2.65 ± 0.860	Monoterpene alcohol	MS, KI
43	16.921	Isothymol methyl ether	1237	1244	_	6.14 ± 0.720	_	Aromatic monoterpenoid	MS, KI
44	17.02	Neral (β-citral)	1240	1240	**34.99 ± 3.530**	_	_	Monoterpene aldehyde	MS, KI
45	17.22	carvone	1247	1248	_	_	**42.5 ± 6.800**	Monoterpene ketone	MS, KI
46	17.35	cis-Geraniol	1252	1254	4.24 ± 0.520	0.39 ± 0.002	_	Monoterpene alcohol	MS, KI
47	17.52	Piperitone	1258	1254	_	0.08 ± 0.003	_	Monoterpene ketone	MS, KI
48	17.7	Carvenone	1263	1258	_	0.01 ± 0.002	_	Methane monoterpenoid	MS, KI
49	17.81	Citronellyl formate	1268	1273	_	0.4 ± 0.030	_	fatty alcohol ester	MS, KI
50	17.94	Geranial	1272	1277	**36.35 ± 4.230**	_	_	Monoterpene aldehyde	MS, KI
51	18.16	Bornyl acetate	1280	1284	_	_	0.1 ± 0.003	Monoterpenes	MS, KI
52	18.38	2-Undecanone	1288	1292	0.08 ± 0.010	_	_	Organic ketone	MS, KI
53	18.47	Carvacrol	1299	1300	_	1.54 ± 0.021	_	Monoterpenoid phenol	MS, KI
54	18.5	Thymol	1292	1292	_	**17.4 ± 3.410**	_	Monoterpenoid phenol	MS, KI
55	18.76	Dihydrocarvenyl acetate	1301	1304	_	_	0.03 ± 0.001	Monoterpenes	MS, KI
56	18.77	Undecanal	1301	1303	0.02 ± 0.001	_	_	Organic aldehyde	MS, KI
57	18.92	Isopulegyl acetate	1307	1335	_	_	0.03 ± 0.004	Monoterpenes	MS, KI
58	19.36	Dihydrocarvyl acetate	1322	1344	_	_	3.97 ± 0.053	Monoterpenes	MS, KI
59	19.43	Geranic acid	1355	1347	0.06 ± 0.002	_	_	Poly unsaturated fatty acid	MS, KI
60	20.02	Citronellol acetate	1345	1355	0.02 ± 0.002	_	_	Sesquiterpenes	MS, KI
61	20.071	Thymyl acetate	1347	1349	_	0.45 ± 0.001	_	Monoterpene	MS, KI
62	20.31	Geranic acid isomer	1355	1355	0.25 ± 0.030	_	_	Poly unsaturated fatty acid	MS, KI
63	20.31	Carvyl acetate	1355	1346	_	_	1.51 ± 0.090	Monoterpenes	MS, KI
64	20.67	Isobornyl propionate	1368	1388	_	0.06 ± 0.003	_	Sesquiterpene	MS, KI
65	20.67	α-copaene	1368	1372	_	_	0.02 ± 0.004	Sesquiterpene	MS, KI
66	20.83	Geranyl acetate	1374	1368	0.77 ± 0.021	_	_	Sesquiterpenes	MS, KI
67	20.93	β-Bourbonene	1377	1385	_	0.15 ± 0.002	0.76 ± 0.050	Sesquiterpene	MS, KI
68	21.1	β-Elemene	1383	1384	_	_	0.29 ± 0.040	Sesquiterpene	MS, KI
69	21.48	Methyl eugenol	1396	1395	1.29 ± 0.310	_	_	Phenyl propanoid	MS, KI
70	21.89	Caryophyllene	1411	1418	0.02 ± 0.004	2.46 ± 0.540	0.46 ± 0.030	Sesquiterpenes	MS, KI
71	22.14	α-copaene	1421	1380	_	0.04 ± 0.002	_	Hydrocarbon	MS, KI
72	22.26	t-α-Bergamotene	1426	1436	0.03 ± 0.001	_	_	Bicyclic monoterpenes	MS, KI
73	22.425	Citronellyl propionate	1433	1444	_	0.01 ± 0.001	_	fatty alcohol ester	MS, KI
74	22.54	Germacrene D	1437	1477	_	0.01 ± 0.002	0.13 ± 0.007	Sesquiterpene	MS, KI
75	22.79	cis-a-Bisabolene	1488	1518	0.02 ± 0.004	_	_	Sesquiterpenes	MS, KI
76	22.79	Humulene	1447	1442	_	0.12 ± 0.006	0.05 ± 0.000	Sesquiterpene	MS, KI
77	23.04	( + )-epi-Bicyclosesquiphellandrene	1457	1452	_	_	0.12 ± 0.020	Sesquiterpene	MS, KI
78	23.241	Geranyl isovalerate	1464	1582	_	0.14 ± 0.003	_	fatty alcohol ester	MS, KI
79	23.34	(Z,Z)-α-Farnesene	1468	1506	_	_	0.01 ± 0.001	Sesquiterpene	MS, KI
80	23.38	γ-Muurolene	1470	1477	_	0.66 ± 0.007	_	Sesquiterpene	MS, KI
81	23.42	Ylangene	1471	1470	_	_	0.02 ± 0.003	Sesquiterpene	MS, KI
82	23.52	Germacrene D isomer	1482	1475	_	_	0.16 ± 0.001	Sesquiterpene	MS, KI
83	23.73	α-Guaiene	1483	1490	_	_	0.03 ± 0.000	Sesquiterpene	MS, KI
84	24.35	γ-Muurolene	1508	1491	_	_	0.1 ± 0.000	Sesquiterpene	MS, KI
85	24.36	γ- Cadinene	1508	1513	_	0.08 ± 0.001	_	Sesquiterpene	MS, KI
86	24.58	Delta- Cadinene	1517	1523	_	0.11 ± 0.000	_	Sesquiterpene	MS, KI
87	24.59	Cis-Calamenene	1517	1531	_	_	0.16 ± 0.000	Sesquiterpene	MS, KI
88	25.93	α-Bourbonene	1569	1531	_	0.02 ± 0.005	_	Sesquiterpene	MS, KI
89	26.01	Spathulenol	1573	1576	_	0.02 ± 0.001	_	Sesquiterpene	MS, KI
90	26.15	Caryophyllene oxide	1578	1577	0.06 ± 0.002	0.97 ± 0.130	0.08 ± 0.001	Sesquiterpene	MS, KI
91	26.9	Eudesmol	1608	1602	_	0.02 ± 0.001	_	Sesquiterpene	MS, KI
92	27.04	Cadinol	1614	1635	_	0.24 ± 0.060	_	Sesquiterpene	MS, KI
Total monoterpenes (%)				97.10		93.04	97.61		
Total sesquiterpenes (%)				0.89		4.92	2.39		
Miscellaneous (%)				1.70		1.97	_		
Total percentage (Approximated %)				100		100	100		

(_): indicate absence of volatile component.

## Data Availability

Data are available with the authors’ reham.kased@bue.edu.eg and dina.elkersh@bue.edu.eg.

## Supplementary Materials

The following supporting information can be downloaded at: https://www.mdpi.com/article/10.3390/life12101587/s1, Table S1: Oils were added with concentrations as shown in the table based on their MIC results. Table S2: Mean readings of colonies count on days 1, 5 and 7. Figure S1: Major identified volatiles in herbs of *Cymbopogon citratus*, *Thymus vulgaris* and *Mentha virdis*.
