# Supplementary material for: GC–MS Profiling of Naturally Extracted Essential Oils: Antimicrobial and Beverage Preservative Actions
Source: Life (Basel). 2022 Oct 12;12(10):1587. doi: 10.3390/life12101587 (PMC9605189; doi:10.3390/life12101587)
Supplement: Supplementary file 1 [file life-12-01587-s001.zip › life-1931693-supplementary.pdf]

Table S1. Oils were added with volumes as shown in the table based on their MIC results.

**Oil (MIC)**	***S. aureus*** **(1 mL)**	***E. coli*** **(1 mL)**	***C. albicans*** **(1 mL)**
MV	50 µL	6.4 µL	12.8 µL
ML	200 µL	0.8 µL	50 µL
limonene	400 µL	6.4 µL	6.4 µL
1,8 cineole	400 µL	200 µL	200 µL

Table S2. Mean readings of colonies count on days 1, 5 and 7.

**Microbe**	**Volatile oil**	**Days count**		
		**Day 1 count**	**Day 5 count**	**Day 7 count**
E. coli	*Mentha V.*	300±10	92±7	< 30
	Mentha L.	300±20	110±15	< 30
	Limonene	300±30	125±10	< 30
S. aureus	*Mentha V.*	300±20	80±8	< 30
	Mentha L.	300±30	150±18	< 30
	Limonene	300±30	180±20	< 30
C. albicans	*Mentha V.*	300±10	90±8	< 30
	Mentha L.	300±15	110±10	< 30
	Limonene	300±25	130±15	< 30

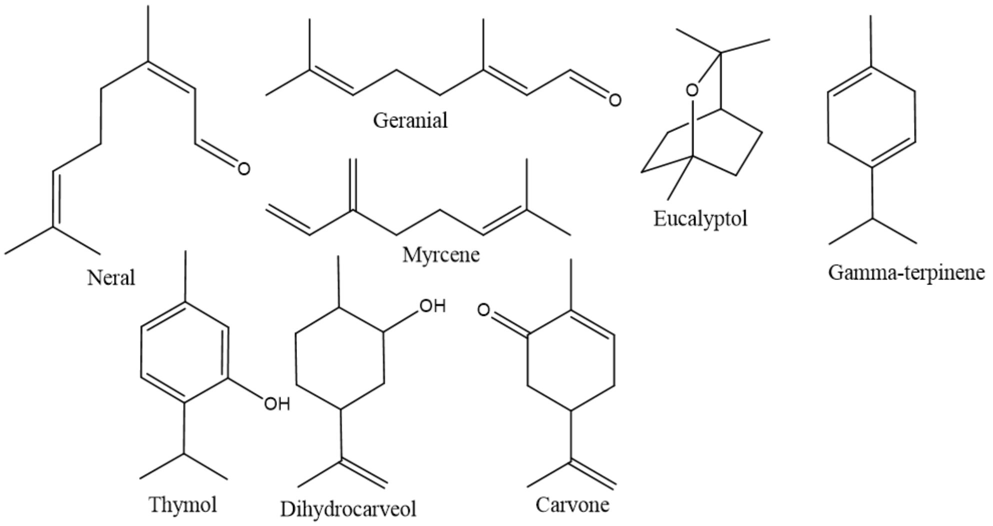

Figure S1. Major identified volatiles in herbs of *Cymbopogon citratus*, *Thymus vulgaris* and *Mentha virdis.*
